# Supplementary material for: Closing the publishing gender gap in economics and political science: Does a critical mass matter?
Source: PLoS One. 2025 May 21;20(5):e0323364. doi: 10.1371/journal.pone.0323364 (PMC12094739; doi:10.1371/journal.pone.0323364)
Supplement: S1 Table — (DOCX) [file pone.0323364.s001.docx]

| Australian National University |
| --- |
| California Institute of Technology |
| Carnegie Mellon University |
| City University of Hong Kong |
| Columbia University |
| Cornell University |
| Duke University |
| ETH Zurich |
| Fudan University |
| Harvard University |
| Johns Hopkins University |
| King’s College London |
| Kyoto University |
| Massachusetts Institute of Technology |
| McGill University |
| Nanyang Technological University |
| National University of Singapore |
| New York University |
| Northwestern University |
| Peking University |
| Princeton University |
| Seoul National University |
| Shanghai Jiao Tong University |
| Stanford University |
| Technical University of Munich |
| The Chinese University of Hong Kong |
| The Hong Kong University of Science and Technology |
| The London School of Economics |
| The University of Edinburgh |
| The University of Hong Kong |
| The University of Manchester |
| The University of Melbourne |
| The University of New South Wales |
| The University of Queensland |
| The University of Sydney |
| The University of Tokyo |
| Tsinghua University |
| UCL |
| University of British Columbia |
| University of California, Berkeley |
| University of California, Los Angeles |
| University of California, San Diego |
| University of Cambridge |
| University of Chicago |
| University of Michigan, Ann Arbor |
| University of Oxford |
| University of Pennsylvania |
| University of Toronto |
| Yale University |
| Zhejiang University |
