# Supplementary material for: Closing the publishing gender gap in economics and political science: Does a critical mass matter?
Source: PLoS One. 2025 May 21;20(5):e0323364. doi: 10.1371/journal.pone.0323364 (PMC12094739; doi:10.1371/journal.pone.0323364)
Supplement: S2 Table — (DOCX) [file pone.0323364.s002.docx]

| Variables | Economics departments | | | | Political science departments | | | |
| --- | --- | --- | --- | --- | --- | --- | --- | --- |
|  | Average | Standard Deviation | Minimum | Maximum | Average | Standard Deviation | Minimum | Maximum |
| All scholars | 34 | 13.9 | 3 | 64 | 30 | 26.1 | 1 | 142 |
| Female scholars | 7 | 4.2 | 0 | 15 | 10 | 10.4 | 0 | 59 |
