# Supplementary material for: Closing the publishing gender gap in economics and political science: Does a critical mass matter?
Source: PLoS One. 2025 May 21;20(5):e0323364. doi: 10.1371/journal.pone.0323364 (PMC12094739; doi:10.1371/journal.pone.0323364)
Supplement: Attachment — Submitted filename: rev_R2.pdf [file pone.0323364.s007.pdf]

Dear Authors,

Thank you for your detailed responses to all comments and efforts in preparing the dataset. I appreciate your thoroughness and have a few clarifying questions and suggestions.

- In Table 1, you are reporting mean values. To enhance clarity, it may be helpful to explicitly state in the title or text that this table represents mean values of the presented indicators. Additionally, I would like to confirm whether the table is based on the following sample sizes: "*For political science, our data included 1,482 scholars, and we retrieved data for 1,718 scholars in economics*." Will these sample sizes remain consistent across all subsequent calculations in the analysis?

- When transitioning to the department level in Figures 1-4, you note: "*Departments with 0 women or with incorrect information for women in our dataset (2 in economics and 5 in political science) are excluded from the analysis*." Please clarify at what stage of the analysis these cases were removed.
  I noticed that your .do-file includes a data correction step where erroneous values are adjusted, for example, for *Anja Faße*. Following this, you compute Table 1. However, in the figure descriptions, you mention additional case exclusions. Were these exclusions limited to the researchers' *name* explicitly addressed in your .do-file, or were other adjustments made? Specifically, I do not see where *Li Jing* from Fudan University was removed in the data. This case lists a female assistant professor with ~77,000 citations in Google Scholar. However, it is most likely a misattribution of citations belonging to *Li Jin,* a male genetics professor from the same university.

  - Was the *Li Jing* case one of the seven excluded cases? Was it included in the calculation of Table 1?
  - Were these cases excluded when computing bibliometric indicators at the departmental level for Figures 1–4 and in the regressions?

  Clarification of this process would be greatly appreciated.

Minor comments:

- The dataset does not include the number of publications used to construct Figure 1.
