# Supplementary material for: Closing the publishing gender gap in economics and political science: Does a critical mass matter?
Source: PLoS One. 2025 May 21;20(5):e0323364. doi: 10.1371/journal.pone.0323364 (PMC12094739; doi:10.1371/journal.pone.0323364)
Supplement: Attachment — Submitted filename: PLOS One Letter 1.docx [file pone.0323364.s008.docx]

Dear Dr. Lovakov,

Thank you very much for allowing us to revise our manuscript thoroughly. We also want to thank the three reviewers for their helpful comments. As per your instructions, I have been very thorough to address reviewer comments 1 and 2, but not 3. Please find below an explanation how we incorporated their comments.

Editor

| Comment | Response |
| --- | --- |
| All data needs to be publicly available | We have added the dataset, codebook, and Stata code with this submission. |

Reviewer 1

| Comment | Response |
| --- | --- |
| The approach used in the article, analyzing the gender gap at the department level, is not commonly seen in bibliometric studies due to its labor-intensive nature. As the authors rightly note, research more frequently focuses on discipline/university/country-level analyses. I would appreciate a more detailed explanation of the advantages of the department-level approach in the text | We added a more detailed description on pages 2 and 3:  Among others we write:  *We build on the scarce, but budding literature, which discusses men and women’s publication performance to detect the existence and the magnitude of the gender gap in publishing. We deviate from this literature in one important aspect. Contrary to existing studies, we do not use one or several publications as the unit of analysis. Rather, we use the individual scholar as observational unit, and then aggregate this information for university departments. We believe that this approach provides more definitive evidence on the existence and the size of the gender gap in academic output in economics and political science. First, analyzing gender gaps at the individual level better aligns with productivity metrics. Second, aggregating at the department level captures the collaborative environments, allowing for a fruitful comparison between disciplines; in our case political science and economics. In more detail, we constructed a novel dataset by web-scraping bibliographical information on all scholars in the economics and political science departments with a Google Scholar Page of the top 50 universities according to the QS Universities Ranking in 2023. Using first names to assign gender, we compare research output and citations of women and men. Our data comprises 1,718 scholars in economics (21 percent women) and 1,482 in political sciences (34 percent women). We establish a gender gap at the individual level in both economics and in political science, the average woman publishes fewer papers and receives fewer citations than the average man.* |
| The text provides a detailed and transparent description of the sampling and data collection process. In addition, more details on the features and limitations of the bibliometric database used would be beneficial. It is important to inform readers about how Google Scholar is formed, including the self-registration of accounts by researchers, how publications are added to a profile, etc. Addressing these points in the text could underscore the limitations of using Google Scholar as a source of bibliometric data for the purposes of this research. | We list on page 12 that the percentage of authors with Google Scholar information in our dataset is 75% in economics and 69% in political science.  We also added some information on page 13 to explain how Google Scholar performs the indexing. We write:  *Once scholars have created their Google Scholar page, all published content by said author is automatically added to her/ his website (for more information, please check Scholastica 2024, and Harzing 2013, Halevi et al. 2017, and Delgado López-Cózar et al. 2019). While limitations exist, such as occasional errors in attribution, double counting of publications—which authors can manually correct—and the inclusion of content without the highest quality control, these issues have been mitigated over time through improvements in automatic algorithms and publisher coverage*. |
| The theoretical framework in the article is exciting, but a more detailed explanation of the mechanism underlying the critical mass theory as applied to the paper’s research question would be appreciated. Why might achieving a certain percentage of women on the faculty correlate with more publications and citations for women in the same department? For instance, one could presume that women might work more comfortably among women, potentially leading to a warmer working environment. Hence, resources that women might otherwise expend on additional communication in more masculine settings could be directed toward research activities. This guess is an example and does not necessarily explain the mechanism at work under critical mass theory in the context of gender gaps in academic publishing, but a more detailed description of the possible mechanisms in the theoretical framework section could be interesting. | We have addressed this point in several parts of the manuscript.  First, in the introduction, we added a clarification of the mechanism:  *According to the concepts of substantive representation and critical mass (Pitkin 1967; Mansbridge 1999; Kanter 1977a,b) women in departments with a high share of female faculty should outperform those in departments with fewer female faculty in terms of publications and citations relative to men’s, as greater representation fosters stronger networks, equitable access to resources, among others.*  Second, added two overview paragraphs in the conceptual framework section, where we explain the concept of critical mass more clearly and delve into the mechanisms (last sentence of the second paragraph). For example, on page 3 we write:  *To assess whether women’s descriptive representation influences their substantive representation in the publication world, we rely on the concept of critical mass. The idea behind critical mass theory is that a group of people, if it is big enough, and if it works together for the collective, can trigger sustained collective action, which, if sustained, can become institutionalized (see Oliver et al. 1985; Grunenbaum 2015). Applied to women in politics, critical mass theory would predict that as more women are included/become present in certain spaces, they often work together or create alliances that influence and change the dominant (male) group, as well as possibly pave the way for more women to be represented in these spaces (Childs & Krook, 2008)*  *Adopting this logic in our context, we expect that a higher numerical presence of women in a department triggers a better performance by each female scholar in publications and citations, more when the level of female representation is higher. That is, the theory of critical mass predicts not only a positive association between female representation and female-favorable outcomes, but also a more accentuated influence the more balanced the representation between men and women becomes. Many empirical studies find support for the prediction that increased numbers of women in politics should increase their chances to pass women-friendly policies increasingly favoring women. (e.g., Studlar & McAllister 2002; Childs and Krook 2009; Kanthak & Krause 2012; Fokum et al. 2020; Mechkova & Carlitz 2021). In our context, multiple mechanisms may generate such an outcome: women tend to favor women without compromising quality (Bransch & Kvasnicka, 2022; Chari & Goldsmith-Pinkham, 2017) and are more likely to collaborate with other women (Ferber & Teiman, 1980; Piscopo et al., 2023; Ductor & Prummer, 2024), potentially fostering a more supportive environment that allows women to focus more effectively on research activities.*  Finally, we speculated about some of the mechanisms that might be in play in explaining why a critical mass of women might trigger some higher publication performance in the conclusion.  *As such our article provides evidence, even if this evidence is only indirect, that increasing the number of women can set in motion processes that favor the inclusion of women in the discipline. For example, Gruenbaum (2015) affirms that achieving a critical mass of women can trigger what he labels ‘interactive innovations’ and ‘reciprocal interdependence’ (p. 142). The former concept refers to the idea that increasing the percentage of women in university departments can create progress in a non-linear fashion through sequential stages. The strong variation in publication output between men and women in various departments with similar percentage of female scholars shows that noy everyone benefits from general progress in the same way. Yet, the discipline seems to have benefited as a whole generating processes of reciprocal interdependence. These processes imply that more women in the discipline not only trigger women to publish more but should also increase the presence of women on editorial boards and as editors. This, in turn, could be a sign of a virtuous cycle of more empowerment. This interpretation comes with the caveat that there needs to be a critical mass of women to set these processes in motion. Due to the low percentage of female scholars in economics’ departments, it seems that these processes have not started or only incompletely started there.* |
| On page 11, it is noted: “...when representation is balanced, we should not see any difference between men and women in publication performance,” which could be a strong statement within the research question posed. It is advisable for the article to mention other factors that could influence the gender gap in publications, even within the same department. For instance, years of work experience and the unequal impact of parenthood on mothers and fathers could be significant factors, considering the cumulative nature of used bibliometric indicators. Other important factors could include gender gaps in grant funding and career promotion, with explanations ranging from direct discrimination and gender stereotype reproduction to behavioral economics perspectives, such as a risk-averse etc. In any case, I believe a discussion of other factors that may influence the gender gap in publications and citations would be useful. | We agree that various factors that exceed female representation can influence gender gaps in academic output. We qualified the phrase alluded by the referee as  *… when representation is balanced, we should move towards equality between men and women in the publication performance—. More broadly, we can expect some synergies; more women in the discipline should increase the percentage of women in influential positions such as editors, editorial board members, and presidents in national and international professional associations. Female scholars should also be more numerous on grant applications and access better funding.*  We did mention other factors on page 7 and 8, We actually believe that the processes set in motion by a critical mass should benefit women in academia beyond publications and help them overcome structural barriers which will end up to level the playing in the future. For example, on page 5 we write:  *More broadly, we can expect some synergies; more women in the discipline should increase the percentage of women in influential positions such as editors, editorial board members, and presidents in national and international professional associations. Female scholars should also be more numerous on grant applications and access better funding.* |
| The article presents a list of universities included in the sample, but more information about the number of observations for each of the 50 universities would enhance comprehension. For example, expanding Table A1 to include "Total scholars" for each of the two disciplines and "% women.» Or it can be a new table with descriptive statistics on the total number of researchers and female researchers for the two disciplines (min, max, mean, SD, median) could be informative. I believe it's crucial for readers to understand the number of observations forming the assessments. If there are 1,718 researchers across 50 economics faculties, averaging 34 per faculty with 21% women, there would be an average of 7 women per faculty. However, universities may vary greatly in terms of overall numbers, and this distribution is important to see, especially when using average values in research. Let's suppose that, on average, there are 34 researchers per faculty; on average, 7 are women and 27 are men. The chance that among 27 men, there will be a researcher-«superstar» who will pull out the average value of bibliometric indicators for men of the whole faculty with his super-high indicators is higher than that such a «superstar» will be among 7 women. I'm not advocating for using median values instead, but readers must clearly comprehend how sensitive the faculty's average bibliometric indicators might be to adding or excluding a single observation, given the total number of observations. | To be as transparent as possible we provided summary information on the size of economics and political science departments, and the number of men and women per department. Supplementary information including minimum and maximums are provided in Table A2.  Regarding the “superstar” effect, we conducted a thorough check for outliers, especially in smaller departments. These checks helped correct some measurement errors.  We write on page 8:  *Before conducting the analysis by university departments, we performed an outlier check, which was particularly important given the small size of some of the departments. During this process, we identified some researchers with incorrect Google Scholar information, which we manually corrected to ensure the accuracy of our data.*  Pertaining to the superstar effect, we also want to reiterate:  While we cannot entirely rule out the influence of the “superstar” effect, we believe it is not inconsistent with the critical mass argument. In fact, the critical mass framework could help explain how increased female representation in a department fosters an environment where women have greater opportunities to excel, with some potentially reaching “superstar” levels. |
| The article uses two groups of bibliometric indicators from Google Scholar—overall indicators and the last six years indicators. The overall indicators reflect a person's career over its entire course. The linkage between overall career achievements and the current department can be quite ambiguous. Perhaps a researcher secured a professor position, let’s say, at Harvard after holding positions at departments with different gender balances and garnered main career achievements outside of Harvard. It’s quite challenging to affirm that the gender balance in this 2023 on Harvard faculty correlates with all cumulative publication achievements of a person. The second group of indicators—the last six years—offers a more accurate timeframe. Therefore, the gender balance in 2023 might be more convincingly related to a researcher’s output in this term. Perhaps viewing bibliometric indicators over even shorter periods could allow for establishing a firmer affiliation with the faculty. It's also worth noting potential concerns about the robustness of the percentage of women on small faculties. The current measure of women’s share in 2023 can be sensitive to hiring one additional person at a small faculty, significantly altering the gender balance. | This is a good point, which we can only address partially.  First, we cannot for whether someone has been at another university during her tenure. However, even if he or she does, she was probably an outperformer. Otherwise, she would not have moved to Harvard.  For the performance over the past two or three years, there is no bibliometric data readily available. We produce the charts for the bibliometric data in the past six years, the most recent information we have, and the results are virtually unchanged (see Figures A1 to A3).  Furthermore, we added a control variable for the percentage of assistant professors. We know that women’s representation should be higher among assistant professors, the category with potentially the least publications and citations. We also control for the proportion of faculty with the title of distinguished professor (see pages 13 and 14). |
| On page 12, it's stated: “...we manually verified the gender of all faculty members in Asian universities.” Does this refer only to Asian universities, or were Asian names in universities from other countries, where such names can occur, also checked manually? | It refers to all faculty members in Asian universities, and faculty with three-letter first names (as a proxy for Asian names which are shorter and more prone to classification errors) in universities from other countries. We clarified this in the text:  *The Gender API first strips the first name from the professors’ full names and uses these to classify them as male or female. Since gender is our key variable in the analysis, we manually verified the gender of all faculty members in Asian universities, those with names with three or fewer letters in other universities (as proxy for Asian-sounding names), and all others with an accuracy index below 80% according to the Gender API, as well as other hand-picked cases. This manual check covered about 90 percent of the names and confirmed an overall classification accuracy of nearly 90 percent.* |
| On pages 13-14, it's noted: “To control for country-specific effects, we add a dummy variable for the US and the UK, with all other countries serving as the reference category. In the US and the UK, the pressure to publish is probably highest, and hence we expect departments in these two countries to have a different pattern in publications and citations (Van Dalen 2021).” More detail is needed on why Australian and Canadian universities are grouped with Asian universities and why their institutional characteristics and publication patterns would be closer, for instance, to Japan than to the UK or US. | We acknowledge the point by the referee and now control for all countries (see all regression models). |
| The term “article” is used several times in the text. Given that the database used comprises a broader list of publications than articles alone, a more general term such as "publications" might be suitable. | We changed the term article in favor of publication throughout the text. |

Reviewer 3

| Comment | Response |
| --- | --- |
| The authors should revise their Methods section in order to provide an accurate report of their research. For instance, the authors state that they “estimate bivariate and multiple regression models linking the proportion of women to the gender gaps in publications and citations (i.e., i10-index, total number of citations, citations per article, and citations of the most cited article)” (p. 13) but only bivariate analyses are presented in the article. | We clarified this. On page 7 and 8, we now explain the statistical procedures we use more clearly. We write:  After establishing the existence of such gender gaps, we display the relationship between female representation and these gaps (the inverse, as previously defined) visually, in scatter plots. In every chart, we include data for both economics and political science, adding locally weighted scatterplot smoothing (Lowess) curves. These non-parametric fits capture non-linear relationships in the data without assuming a specific functional form.  Next, we estimate departmental-level regression models linking the proportion of women to the gender gaps in publications and citations (i.e., i10-index, total number of citations, citations per publication, and citations of the most cited publication). We include a dummy for political science departments, and the interaction of the proportion of women with a dummy for to capture differences in the slope. We add several control variables. For example, we control for the percentage of assistant professors in a department. Assistant professors are the category where women’s representation is the highest among all academic (see also: Santos & Dang Van Phu, 2019). However, scholars at the assistant professor level are also the category with potentially the least publications and citations of all ranks. We also control for the proportion of distinguished professors. Together, these two variables help capture the academic rank structure of the department, which may also influence the relationship between female representation and the gender gap. Finally, we control for country-specific effects and add a dummy variable for all countries. We believe that in the US and the UK, for instance, the pressure to publish is probably highest, and hence we expect departments in these two countries to have a different pattern in publications and citations (see Fanelli 2020; Van Dalen 2021). |
| The distribution of the observations as depicted in Figures 1-4 and the R2 values reported suggest that a linear relationship is not the best functional form to model the relation between the variables. The authors should therefore provide a justification for their adoption of this model or explore the results of alternative modeling techniques. | This point is well taken. We have replaced the linear regression lines in Figures 1–4 with lowess (locally weighted scatterplot smoothing) curves, a non-parametric method that provides a more flexible fit to the data. This approach allows us to visually capture the non-linear relationship between female representation and gender gaps without imposing a specific functional form. The updated figures reveal a steeper slope at higher levels of female representation, supporting the idea that reaching a critical mass significantly reduces gender gaps. This adjustment addresses the limitations of a linear model and aligns with the critical mass framework discussed in the paper. |
| The authors' justification for the inclusion of a dummy variable only in the cases of US and UK is not clear. They argue that in these countries “the pressure to publish is probably highest” (p. 14) and cite an article addressing the perceived work pressure in Dutch Universities. They should improve their arguments or include dummy variables for all countries. | In the revised version, regressions include dummies for all countries. |
| The authors keep departments with no women in their analysis, assigning 0 to the inverse gender gap in these cases. The justification for the imputation of this value is not clear, and I would suggest removing these cases from the analysis or replacing them with other departments (namely, the departments in the next universities in the rank), as they are qualitatively different from the rest of the cases and may be introducing distortions. | We removed departments with no women with valid Google Scholar information from the analysis. |
| I would suggest including Table A2 in the Main text, as it provides relevant contextual information. Moreover, information in this table is currently presented in an inconsistent manner: authors should include absolute values for Men and Women both for overall and for the last six years and include all variables in both cases (or justify their absence). | We followed your suggestion. We have included Table A2 in the main text, now Table 1, to provide the relevant contextual information. Additionally, we have updated the table to present absolute values for both men and women for overall differences and differences between the last six years, ensuring consistency across all variables. When it comes to citations for the most cited publication in the last six years, we have added a note explaining that this indicator is only available for the entire publication history. |
| This methodology excludes non-binary identities and may lead to misgendering, as it does not rely on self-identification. Authors should state this limitation. Furthermore, authors should revise their use of the terms “female”/“male” and “women” ”/“men” and adopt a consistent criterion. | We state on page 10 that “this coding excludes non-binary identities and may lead to misgendering, as it does not rely on self-identification”  We also state on page 10 that we use male and female, and woman and man interchangeably. |
| The authors infer gender relying on algorithmic and manual analysis of researchers’ names.  The relationship between substantive representation—“the congruence between representatives’ actions and the interests of the represented”—and “assessing whether women can publish and receive citations on par with men” (p. 10) is not self-evident, but the authors fail to clearly explain it. | We classified this statement. On page 4, we write:  *Normally, studies discussing the link between the two concepts look at whether women represent women’s interests in policy fields such as reproductive rights, family legislation, spending priorities or environmental legislation (Hessami & da Fonseca 2020; Ramstetter & Habersack, 2020).* |
| While the values for the Betas are reported with one decimal in Figures 1-4, they are rounded in Table 1, authors should use the same number of decimal places in both cases. | In the revised version, we removed the coefficients of the linear fit as we included a non-linear fit (lowess). |
| The authors state that all data underlying the findings described in their manuscript will be fully available without restriction, but at the moment of this review it is not yet available.  ________________________________________ | We are submitting the data with the revised version. |
