# Supplementary material for: Closing the publishing gender gap in economics and political science: Does a critical mass matter?
Source: PLoS One. 2025 May 21;20(5):e0323364. doi: 10.1371/journal.pone.0323364 (PMC12094739; doi:10.1371/journal.pone.0323364)
Supplement: Attachment — Submitted filename: PLOS One Letter_rev.docx [file pone.0323364.s009.docx]

Dear Dr. Lovakov,

Thank you very much for allowing us to revise our manuscript thoroughly. We also want to thank the reviewer 1 for their helpful comments. We have been very thorough to address yours and reviewer 1’s comments. Please find below an explanation how we incorporated their comments.

Editor

| Comment | Response |
| --- | --- |
| One of your descriptions of Google Scholar may not be entirely accurate (namely "Once scholars have created their Google Scholar page, all published content by said author is automatically added to her/ his website"). As far as I know, Google Scholar adds articles to the profile when the "add articles automatically" option is selected. I did not find any other information about this in the references you cited. Could you please check and confirm this? | Thank you for raising this point—your observation is correct. In practice, we believe that automatic update is the dominant strategy scholars use due to its convenience and low cost. Nonetheless, we have revised the text to explicitly acknowledge that not everyone might choose this option:  *We think that Google Scholar constitutes a good source for bibliographic information at the individual level. It is the largest and most comprehensive search engine displaying academic content [40], indexing a broad range of academic content such as journal articles, working papers, books and book chapters, conference proceedings, and policy reports, across all disciplines, from all countries, and in all languages. Once scholars have created a Google Scholar profile, they can choose to have It automatically indexes published content such as journal articles, book chapters, and books across all disciplines, from all countries, and in all languages. Once scholars have created their Google Scholar page, all published content by said author is automatically added to her/ his website, or they can opt for manual input. Even when manual input is selected, Google Scholar’s algorithms automatically identify and suggest potential publications by the author, and can notify scholars if this option is enabled (for more information, please check [11, 25, 27, 51]). While limitations exist, such as occasional errors in attribution, double counting of publications—which authors can manually correct—and the inclusion of content without the highest quality control, these issues have been mitigated over time through improvements in automatic algorithms and publisher coverage.* |

Reviewer 1

| Comment | Response |
| --- | --- |
| In Table 1, you are reporting mean values. To enhance clarity, it may be helpful to explicitly state in the title or text that this table represents mean values of the presented indicators. Additionally, I would like to confirm whether the table is based on the following sample sizes: *"For political science, our data included 1,482 scholars, and we retrieved data for 1,718 scholars in economics."* Will these sample sizes remain consistent across all subsequent calculations in the analysis? | The title of Table 1 changed to: *“Table 1. Summary statistics (average values) and gender gaps (average for women divided by average for men) in research output and impact, overall and in the last six years.”*  We added the number of observations, and a mention in the table note: *The number of observations for average citations per publication is smaller than for the remaining indicators due to scholars with 0 publications.*  Also, for reasons that will become clear in our response to the next comment—briefly, the unification of the cleaning process—the sample sizes changed slightly, without affecting the results. |
| When transitioning to the department level in Figures 1-4, you note: *"Departments with 0 women or with incorrect information for women in our dataset (2 in economics and 5 in political science) are excluded from the analysis."* Please clarify at what stage of the analysis these cases were removed. I noticed that your .do-file includes a data correction step where erroneous values are adjusted, for example, for *Anja Faße.* Following this, you compute Table 1. However, in the figure descriptions, you mention additional case exclusions. Were these exclusions limited to the researchers’ *name* explicitly addressed in your .do-file, or were other adjustments made? Specifically, I do not see where *Li Jing* from Fudan University was removed in the data. This case lists a female assistant professor with ~77,000 citations in Google Scholar. However, it is most likely a misattribution of citations belonging to *Li Jin,* a male genetics professor from the same university.   - Was the *Li Jing* case one of the seven excluded cases? Was it included in the calculation of Table 1? - Were these cases excluded when computing bibliometric indicators at the departmental level for Figures 1–4 and in the regressions?   Clarification of this process would be greatly appreciated. | We appreciate the referee’s careful examination of the reproduction package. The referee is right that the cleaning process could be improved, and we have taken this suggestion seriously. We have now unified the cleaning process from the outset to ensure consistency throughout the paper. The modifications are as follows (importantly, they did not meaningfully affect the results):   1- We excluded salient cases of misattributed Google Scholar profiles, such as that of *Li Jin*. While these were already excluded from the correlation analysis, they had not been excluded from Table 1. They are now consistently excluded throughout the analysis. 2- We accurately corrected the information of *Anja Faße*, which had previously been affected by encoding issues when re-opening the data. We identified the problem thanks to the referee’s comments and have now fixed it.   The text has been adjusted accordingly. In the *Scraping bibliographic information from the Google Scholar author page*s section, it now reads:  *An outlier check yields the detection of six errors of misattributed Google Scholar profiles, which were manually corrected. We retrieved data for 1,715 scholars in economics across 50 departments, averaging 34 faculty per department split in 27 men and 7 women, on average, per department. For political science, our data included 1,480 scholars, averaging 30 people per department (i.e., 20 men and 10 women per department) (see S2 Table).*  We also revised the figure notes to exclude the cleaning process. For example, the note for Fig 1 now reads:  *Departments with 0 women in our dataset (2 in economics and 5 in political science) are excluded from the analysis.* |
| The dataset does not include the number of publications used to construct Figure 1. | The number of publications refers to the i10-index. We now added a clarification in the *Scraping bibliographic information from the Google Scholar author page*s section:  *The variables collected for each scholar include the i10-index, which measures the number of publications with at least ten citations, the total number of citations, the citations per publication calculated as citations divided by i10-index, and the number of citations for the most cited publication. Throughout the paper, we use i10-index and number of publications interchangeably.* |
